# Perovskite sensitized 2D photodiodes

**DOI:** 10.1038/s41377-023-01187-2

**Published:** 2023-06-06

**Authors:** Dong Li, Anlian Pan

**Affiliations:** Key Laboratory for Micro-Nano Physics and Technology of Hunan Province, State Key Laboratory of Chemo/Biosensing and Chemometrics, Hunan Institute of Optoelectronic Integration, College of Materials Science and Engineering, Hunan University, Changsha, China

**Keywords:** Optoelectronic devices and components, Integrated optics

## Abstract

A new type of perovskite sensitized programmable WSe_2_ photodiode is constructed based on MAPbI_3_/WSe_2_ heterojunction, presenting flexible reconfigurable characteristics and prominent optoelectronic performances.

The emergence of two-dimensional semiconductors (2DSCs) provides a rich material foundation for the construction of new optoelectronic devices^1,2^. Transition metal dichalcogenides (TMDs), as a representative, has been widely used in the construction of electrically tunable light emitting diodes (LEDs)^3^, gate-controlled p-n junction diodes^4^, and solar cells^5^. However, due to atomically thin geometry of monolayer TMDs, effectively tuning charge doping type and improving light absorption efficiency of them has always been a huge challenge, which greatly limits their application in high-performance optoelectronic devices^6^.

Electrostatic doping opens an effective way to regulate charge carriers in nanomaterials. Compared with traditional lattice doping with impurity atoms, electrostatic doping has been widely concerned and applied in low dimensional nanomaterials without introducing impurity atoms and degrading the intrinsic electronic properties of the nanoscale materials. For example, Lee et al.^7^ demonstrated a programmable device based on reversible solid-state doping of WSe_2_. By manipulating silver ions in solid-state superion silver iodide (AgI), the carrier types of WSe_2_ were adjusted to achieve reverse programming of transistors, diodes, photodiodes, and logic gates. Li et al.^4,8^ achieved high-performance reconfigurable p-n junctions and FET devices by precisely controlling the type of carriers in local floating gates, proving that electrostatic doping can effectively regulate the carrier type and density in TMDs.

One promising way to overcome the low light absorption efficiency of TMDCs is to integrate TMDs with organic dye molecules with high light absorption efficiency^9^. The hybrid lead halide perovskite (LHP), represented by Methylammonium lead iodide (CH_3_NH_3_PbI_3_ or MAPbI_3_), is a good choice as the building blocks of optoelectronic devices due to their high light absorption coefficients, long carrier diffusion lengths, strong defect tolerance, and tunable band structures. However, the “soft lattice” ionic LHPs is usually troubled by ion migration under voltage bias, resulting in poor material stability^10^ and large hysteresis in the voltage-dependent photocurrent^11^. Although undesirable for stable operation of solar cell applications, the migration of positively or negatively charged ions could induce ion accumulation or ionic charge imbalance under applied electric fields^12^, which can be potentially used to reversibly dope 2DSCs, thus to produce photodiodes.

In the regard, writing in this issue of eLight^13^, Xiangfeng Duan, Yu Huang, Dae Joon Kang, and colleagues report a method by exploiting ionic charge imbalance in LHPs to induce reversible doping in 2DSCs, achieving a perovskite sensitized programmable WSe_2_ photodiode based on MAPbI_3_/WSe_2_ heterojunction with flexible reconfigurable characteristics and ultrahigh open circuit voltage. The device shows a switchable open circuit voltage up to 0.78 V and a high EQE of 84.3%. By integrating tunable graphene contacts, the photodiode performance can be further improved to achieve a highest open circuit voltage (*V*_OC_) of 1.08 V and a maximum EQE of 91.3%, greatly exceeding those achieved previously in 2DSC lateral diodes.

In this work, the authors present a WSe_2_/MAPbI_3_ device, where the schematic view and working mechanism of device are shown in Fig. 1. Under high temperature, ions in MAPbI_3_ can be controlled by an external electric field for directional movement. After being frozen at low temperature, these migrated ions are solidified and form ion gates with specific electric field directions in MAPbI_3_, thereby forming non-destructive and reversible electrostatic doping in nearby two-dimensional materials. Based on this working mechanism, the authors can perform controllable and reversible doping in WSe_2_, achieving carrier inversion and p-n junction reconfiguration in the identical device.

**Fig. 1:**
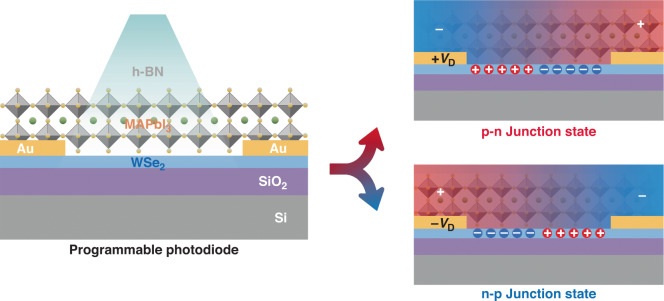
Schematic view of MAPbI_3_/WSe_2_ device structure and working mechanism of the programmable perovskite sensitized WSe_2_ photodiode

Photodiode applications are further explored based on the programmed diode. Under 532-nm laser excitation (15 W/m^2^), the negatively poled photodiode delivered an *V*_OC_ of −0.78 V, and the identical photodiode showed a *V*_OC_ of +0.75 V after the positive lateral poling process. The results demonstrate that the p-n junctions can be reversibly programmed by the electrical poling process, which makes up the shortcomings of traditional silicon-based p-n junctions with fixed performances. Meanwhile, the extracted EQE of 84.3% at 532 nm represents the highest value achieved from lateral or sensitized 2D diode at zero bias voltage^14^. By integrating tunable graphene contacts, the photodiode performance can be further enhanced to a *V*_OC_ of 1.08 V and a maximum EQE of 91.3%. Such strong enhancement indicating that MAPbI_3_ perovskite can served as not only programmable ionic dopants but also the excellent optical absorber and highly efficient sensitization layer.

The unique design of MAPbI_3_/WSe_2_ device provides a new idea to fabricate high-performance programmable photodiodes. In addition, the combination of atomic thin 2D materials and ionic solids enables effective coupling between electronic transport and ionic transport, which may open up a new pathway for unconventional computing, information storage systems, and programmable optoelectronic devices.
